# Effect of Sinetrol^®^ Xpur on metabolic health and adiposity by interactions with gut microbiota: a randomized, open label, dose–response clinical trial

**DOI:** 10.1186/s12986-024-00851-7

**Published:** 2024-10-16

**Authors:** Jananee Muralidharan, Cindy Romain, Linda Chung, Pedro Alcaraz, Francisco Javier Martínez-Noguera, Mayoura Keophiphath, Benjamin Lelouvier, Patricia Ancel, Benedicte Gaborit, Julien Cases

**Affiliations:** 1 Fytexia, ZAE via Europa—3 rue d’Athènes, 34350 Vendres, France; 2 Research Center for High Performance Sport—UCAM Universidad Católica de Murcia, Murcia, Spain; 3 DIVA Expertise, Centre Pierre Potier, 1 place Pierre Potier, 31100 Toulouse, France; 4 VAIOMER, 516 Rue Pierre et Marie Curie, 31670 Labège, France; 5 INSERM, INRA, C2VN, Aix Marseille Univ, Marseille, France

**Keywords:** Obesity, Polyphenols, Gut microbiota, Fat loss, Metabolism

## Abstract

**Background:**

Sinetrol^®^ Xpur is a polyphenolic ingredient rich in citrus flavonoids that has shown weight loss effects in previous studies. The dose dependent nature, gut microbial actions of this product has not been explored previously, thus presented in this study.

**Methods:**

In this open label study, we evaluated the effect of Sinetrol^®^ Xpur supplementation on healthy but overweight/obese adults (20–50 yrs) for 16 weeks. Participants (n = 20) were randomly allocated to a high dose group (HD, 1800 mg/day) or low dose group (LD, 900 mg/day) of the product for 16 weeks. Fat composition, gut microbial composition, were evaluated using MRI and 16S rDNA sequencing respectively at week 1 and 16.

**Results:**

We observed HDL, HbA1C, LDL and leptin improved significantly over 16 weeks, irrespective of the dosage. There was a trend for decrease in visceral adipose tissue (VAT), BMI over time and body weight displayed a trend for dose dependent decrease. *Eubacterium xylanophilum*, *Ruminococcacea UCG-004* genus which increased in HD and LD respectively were negatively associated to VAT. Both doses increased butyrate producers such as *Eubacterium ruminantium* and *Ruminococcaceae NK4A214* genus.

**Conclusions:**

Overall chronic supplementation of Sinetrol^®^ Xpur, irrespective of their dose improved HDL, HbA1c, LDL and leptin and tended to decrease visceral adipose tissue via changes in gut microbiota.

*Trial registration number* NCT03823196.

**Supplementary Information:**

The online version contains supplementary material available at 10.1186/s12986-024-00851-7.

## Introduction

Prevalence and increase in the global obesity epidemic have led to the development of several prevention and treatment strategies. Lifestyle modifications such as consuming balanced diet and increasing physical activity are effective methods, although adherence to these approaches is poor [1]. Amongst other strategies, the use of plant based polyphenolic compounds have been increasingly recognized for their ability to promote weight loss and provide cardiovascular protection. Polyphenols have also shown to act directly on adipose tissue which serves as an important site in the progress of metabolic disorders [2].

Ingested polyphenols undergo a wide range of enzymatic modifications in the small intestine resulting in phenolic metabolites which in turn are absorbed and distributed to various body sites before being excreted. However, only 5–10% of the polyphenols undergo this fate, whereas the rest reaches the colon where they are bio-transformed by actions of several microbial enzymes [3]. The resulting metabolites, referred to as colonic metabolites are either excreted via feces or absorbed via portal circulation where they undergo phase-II biotransformation after which they are circulated to the organs and excreted in the urine [4]. Prior studies have shown that accumulation of polyphenolic metabolites in organs such as the liver, the adipose and the brain could indicate their possibility of action at these sites [5–7]. Especially in the adipose tissue, these compounds have shown to exhibit their effects via various mechanisms such as promoting gene expression of Ucp1, Chrebp, Glut4 and reducing activities of NF-kB, JNK pathway [8, 9]. Flavanones such as naringenin and hesperidin have shown glucose lowering and lipid modulating effects in invitro studies, however the human studies have shown inconsistent results [10, 11]. High interindividual variation in the gut microbial metabolism of polyphenols are attributed as one of the reasons for these inconsistent results. Studies by Assini et al. and Mena et al. have shown that various individuals display a wide distinction in the presence of polyphenolic colon metabolites thus indicating a potential difference in their bioavailability and bioactivity [8, 12, 13].

The important role of gut microbiota in energy homeostasis and metabolic regulation is well established. The interaction of polyphenols with gut microbiota is bidirectional and complex. The aglycone release by colonic microbiota is the rate-limiting step in the absorption of naringenin [14]. Thus, understanding the changes in gut microbiota is an important part of elucidating the mechanism of action of the polyphenols. Members of genera such as Eubacterium have been associated with metabolism of flavonoids, whereas other species such as *Flavonifractor plautii* and *Enterococcus casseliflavus* have shown to involve in metabolism of flavonoid such as quercetin [15]. Other bacterial genera such as *Lactobacillus* and *Bifidobacterium* have also shown to increase with polyphenol consumption [16]. Interestingly members of Bifidobacteria have also been inversely associated with adipose tissue inflammation, glucose tolerance and lipopolysaccharide levels, thus potentially indicating an indirect effect of polyphenol consumption [17, 18].

Although many studies indicate a strong effect of polyphenol consumption on body composition and gut microbiota interactions, only few of the studies evaluated these effects during chronic consumption in humans. Taking this into account, in this study we report the effect of chronic consumption (16 weeks) of two doses of Sinetrol^®^ Xpur on weight loss, improvement of metabolic health and corresponding changes in gut microbiota. Sinetrol^®^ Xpur is a patented polyphenol extract rich in flavanones from grapefruit, pomelo, sweet orange, blood orange and caffeine derived from guarana extract. Previous studies on Sinetrol^®^ Xpur have shown a reduction in body weight (− 3.28 ± 0.24%) and body fat percentage (− 9.73 ± 0.54%) in comparison to placebo group [19, 20]. However, the possibility of improving the bioactivity could be dose dependent which we have explored in this study. We also report the gut microbiota interactions between Sinetrol^®^ Xpur and its two doses that could be involved in its mechanism.

## Methods

### Study design, product, and participants

This study reports the results from an open labelled clinical trial with 20 overweight (BMI 27–30 kg/m^2^) but otherwise healthy volunteers in the age group of 20–50 years. Participants also were evaluated for their total fat mass using Bioelectrical impedance analysis (BIA) assessment with criteria of ≥ 25% for men and ≥ 32% for women. Participants declared of no known existing chronic disease conditions. The exclusion criteria included presence of any metabolic/chronic diseases, current use of medication/food supplements, formerly obese with a history of yoyo-effect, pregnancy/lactation/menopause in women and any known allergic reactions to components of the supplement (ie orange, grapefruits, guarana and or coffee). All the participants provided a consent to participate form with written signature. Ethics approval was obtained from the Ethical Committee of Universidad Catolica San Antonio de Murcia in compliance with the guidelines laid out in the Declaration of Helsinki and with Good Clinical Practices defined in the ICH Harmonized Tripartite Guideline, CE5551.

The participants were randomly allocated to the high or low dose group consuming either 900 mg/day or 1800 mg/day of the product respectively for everyday during a period of 16 weeks. Randomization of participants into the groups were conducted with Microsoft® Excel program. The product supplemented was Sinetrol^®^ Xpur, a polyphenolic ingredient mainly composed of flavanones. The characteristics and the pharmacokinetics of the product is described elsewhere [21]. Participants were asked to restrain from foods rich in polyphenols (list in Supplemental Information) and were instructed by a dietician to consume normocaloric diet according to their individual needs (calculated from Harris-Benedict equation). They were also advised to not perform any increased physical activity (measured with pedometer) throughout the duration of the study. Compliance to the study was evaluated by retrieving the pill boxes at the end of study and to count the number of pills left. Compliance to the dietary recommendations were assessed through 24-h diet recall questionnaire (2 interviews during the week and 1 interview during weekend) performed during each visit.

### Anthropometric and biochemical variables

#### Body weight

At each visit to the Research Center, the following variables were monitored: height, body weight on a scale, waist & hip circumferences using a non-stretchable tape. Subjects were in a fasted state since the dinner of the previous day (taken around 8:00 pm) and wearing light clothing and no shoes.

#### Abdominal fat composition

At week 1 and 16, abdominal fat composition was measured using MRI on vertebral endplate of L4/L5 with slice thickness of 2.5 mm. Subsequently images were analysed to estimate the visceral and subcutaneous adipose tissue.

#### Blood sampling

At W1 and W16, with fasting volunteers, 15 mL blood was extracted from the basilica vein with TBD tubes (% mL) Terumo Venoject (Terumo, Leuven, Belgium) with EDTA or dry tube. The blood was centrifugated at 30,000 r.p.m. for 10 min at 4 °C. Immediately after centrifugation, plasma (from EDTA tube) or serum (from dry tube) were extracted and proportionally divided in aliquots of 0.5 mL (Eppendorf tubes). These samples were frozen for further analysis. Various markers were analysed with ELISA assays (1) sTNFR1, sTNFR2, CD14, TNF, IL10, MCP1 [Bio-techne, USA], (2) Zonulin [IDK Diagnostics, Germany], (3) Adiponectin, Leptin [TECO, Switzerland], (4) IL6 [Fujirebio, Japan], were analysed with standard ELISA assays. Free fatty acid [Diasys, Germany] was quantified with calorimetry and hsCRP [Beckman Coulter, USA] with nephelometer.

### Identification and quantification of blood and urine metabolites

Blood sampling was conducted at baseline and week 16 at various time points including baseline (T0), and at 2, 5, 6, 7, 10, 15 and 24 h after Sinetrol^®^ Xpur consumption. Solid phase extraction method was used to extract plasma and samples were stored at -80 C until further analyses. Urine were collected both at week 1 and week 16 at time point 0 and at different collection periods within 0–5 h, 5–10 h, 10–15 h, 15–24 h and 24–48 h. Urine samples were prepared as previously reported by Brindani and colleagues [22]. Briefly, urine samples were defrosted, vortexed, diluted in 0.1% formic acid in water (1:3, v/v), and centrifuged at 14,000 rpm for 10 min. Finally, urine samples were filtered (0.45 μm nylon filter) prior to the UHPLC/ESI–MS/MS analysis.

Urine samples were analysed by a UHPLC DIONEX Ultimate 3000 equipped with a triple quadrupole TSQ Vantage (Thermo Fisher Scientific Inc., San José, CA, USA) fitted with a heated-ESI (H-ESI) (Thermo Fisher Scientific Inc., San José, CA, USA) probe. Plasma samples were analysed by a UHPLC-ESI–MS/MS analysis using the same analytical method used for urine samples, looking for the same flavanone metabolites (about 130, based on available data on human and microbial metabolism of flavanones).

Quantification was performed with calibration curves of standards, when available or using the most structurally similar compound. Data processing was performed using Xcalibur software (Thermo Scientific Inc., Waltham, MA, USA). Further details are provided in the previously published article [21].

### Fecal sampling and 16S rRNA sequencing

Participants were supplied with fecal collection kit including cooling bag, ice pad, sterile collection tube, storage container, and anaerocult plate (VWR, USA). Participants were instructed to collect the faecal samples only after emptying their bladder. Faeces collected was instructed to be bought to the UCAM with the ice pack and cooling bag within 24 h of excretion. Samples were immediately stored at -80 C until further analyses.

Microbial DNA was extracted using QIAamp Fast tool Kit (Qiagen, Hilden, Germany) and DNA concentrations were quantified using UV spectroscopy (Nanodrop®, Thermo Scientific). Bacterial populations were determined using next generation high throughput sequencing of variable regions V3-V4 of the 16S rRNA gener (using Vaiomer universal 16S primers) as described before [23]. The detection of the sequencing fragments was performed with the MiSeq Illumina technology using the 2 × 300 paired end MiSeq kit V3. The targeted metagenomic sequences from microbiota were analyzed using the bioinformatics pipeline established by Vaiomer from the FROGS guidelines [24]. Briefly, after demultiplexing of the bar-coded Illumina paired reads, single read sequences are cleaned and paired for each sample independently into longer fragments. Operational taxonomic units (OTUs) are produced via agglomerative, unsupervised (de novo) single-linkage clustering and taxonomic assignment is performed in order to determine community profiles.

The following specific filters have been applied for this analysis in order to obtain the best results:

- The last 10 bases of reads R1 were removed.
- The last 10 bases of reads R2 were removed.
- Amplicons with a length < 350 nt or a length > 500 nt were removed.
- OTUs with abundance lower than 0.005% of the whole dataset abundance were removed.

The taxonomic assignment was given by Blast + v2.2.30 with databank Silva 132 Parc. We aggregated the samples at genus level and applied a filter for keeping microbiota with relative abundance of > 0.1% and detected in at least 10% of the samples.

### Statistical analysis

Participant anthropometric data were represented as median and standard deviation. We tested for confounders such as diet intake and the pedometer readings. For blood and body weight variables a linear mixed model accounting for time and dose was conducted. Post hoc test was conducted to identify the differences within the group. For microbiota analysis, a negative binomial regression was used from the package NBZIMM in R. Unexpectedly, the carbohydrate and lipid % varied significantly during the study duration (tested by Anova). Hence, they were accounted for within the mixed model. With the abundance at various dose and time, we constructed a heat plot parallel to the results obtained in NBZIMM. We further evaluated the changes (delta) of significantly varying markers and microbiota (CLR transformed) and conducted an association analysis using general linear model, adjusted for sex, dose and other confounders. These are represented in Table 2.

## Results

Twenty participants were included in the study of which nineteen were included in this analysis. One of the participants was removed due to noncompliance. Participants were aged 32.7 (9.6) years in LD and 28.6 (6.6) years in HD. At the beginning of the study, none of the study parameters measured differed between the two groups. No adverse events were reported during the study.

Although participants did not change their total energy consumption during the study, carbohydrate % (LD = 8.3 (10.5), HD = 7.3 (12.1)) and fat % (LD = 6.3 (9.2), HD = 6.9 (13.9)) changed significantly over 16 weeks, without differences between the groups (Additional file 1). To account for these changes, further models were adjusted with these two factors.

### Changes in body weight and body fat (Table 1)

**Table 1:** Characteristics of the study population during the period of 16 weeks

	Low dose		High dose				
	Baseline	Week 16	Baseline	Week 16	Dose	Time	Dose*Time
n	10	10	9	9	*p*-value		
Age	32.7 (9.6)		28.6 (6.6)				
Sex (% Male)	40		66.6				
Weight (Kg)	83.12 (9.84)	83.18 (11.68)	87.83 (6.09)	84.74 (7.00)	0.46	0.06	0.06
BMI (Kg/m2)	28.67 (0.89)	28.63 (1.25)	28.83 (1.26)	27.81 (1.62)	0.53	0.07	0.11
Waist circumference (cm)	88.3 (7.75)	87.1 (7.07)	91.6 (6.65)	89.6 (8.52)			
Glucose (mg/dL)	89.70 (5.40)	91.23 (18.61)	88.77 (9.08)	85.51 (9.68)	0.44	0.83	0.49
HbA1c (%)	5.06 (0.27)	4.78 (0.25)^a^	5.01 (0.22)	4.78 (0.20)^a^	0.81	**< 0.001**	0.45
Total-Cholesterol (mg/dL)	173.54 (30.51)	178.17 (31.99)	185.92 (33.21)	180.50 (24.01)	0.57	0.98	0.44
HDL-Cholesterol (mg/dL)	54.29 (13.71)	68.21 (8.59)^a^	59.39 (12.22)	73.10 (6.36)^a^	0.26	**< 0.001**	0.97
LDL-Cholesterol (mg/dL)	100.98 (25.15)	91.18 (26.80)	115.20 (29.60)	93.49 (21.18)	0.41	**0.04**	0.42
TG (mg/dL)	91.38 (40.86)	94.02 (25.38)	56.67 (17.23)	69.49 (19.50)	0.02	0.18	0.41
hsCRP (mg/L)	3.58 (7.30)	5.03 (6.35)	3.17 (4.31)	2.26 (2.65)	0.5	0.74	0.27
Free fatty acids (mmol/L)	0.34 (0.13)	0.43 (0.28)	0.33 (0.12)	0.37 (0.12)	0.6	0.12	0.57
Zonulin (ng/mL)	28.36 (9.86)	33.04 (10.79)	25.99 (9.92)	25.98 (13.51)	0.27	0.38	0.34
Adiponectine (μg/mL)	8.30 (5.54)	7.91 (3.27)	10.65 (6.05)	6.61 (2.49)	0.73	0.17	0.22
Leptine (ng/mL)	40.78 (31.68)	15.88 (10.86)	32.21 (31.54)	16.96 (14.84)	0.64	**0.02**	0.56
sTNFR1 (ng/mL)	1373.37 (230.50)	1255.23 (191.33)	1300.47 (187.35)	1245.81 (244.44)	0.63	**0.04**	0.33
sTNFR2 (ng/mL)	2754.24 (654.95)	2638.34 (477.62)	2632.08 (520.48)	2660.40 (463.42)	0.81	0.73	0.6
CD14	1233.38 (230.98)	1434.40 (323.01)	1456.89 (390.66)	1480.50 (344.81)	0.32	0.14	0.25
TNF (pg/ml)	0.49 (0.24)	0.50 (0.16)	0.54 (0.33)	0.49 (0.25)	0.85	0.6	0.3
IL6 (pg/ml)	1.73 (0.79)	2.30 (1.45)	2.01 (1.31)	1.41 (0.76)	0.4	0.96	0.13
IL10 (pg/ml)	7.57 (5.18)	7.26 (6.15)	14.08 (8.87)	11.20 (8.47)	0.1	0.2	0.33
MCP1 (pg/ml)	432.69 (211.36)	472.18 (204.38)	425.02 (133.36)	442.02 (211.87)	0.82	0.41	0.77
Adiponectine/Leptine	0.29 (0.18)	0.68 (0.49)	0.67 (0.70)	0.68 (0.51)	0.27	0.22	0.26
Visceral adipose tissue (cm2)	82.92 (23.72)	79.16 (21.99)	87.08 (39.29)	79.81 (29.05)	0.85	0.06	0.47
Sub cutaneous adipose tissue (Deep) (cm^2^)	186.99 (48.29)	187.55 (42.30)	167.54 (27.20)	155.19 (35.25)	0.15	0.29	0.24
Sub cutaneous adipose tissue (Superficial) (cm^2^)	182.43 (54.09)	181.91 (49.97)	166.94 (51.24)	165.32 (47.97)	0.47	0.81	0.96
Total sub cutaneous adipose tissue (cm^2^)	369.42 (75.47)	369.46 (59.95)	334.48 (57.68)	320.51 (62.74)	0.13	0.47	0.55
Pedometer	7013 (2373)	6804 (2480)	7673 (2655)	7121 (1561)	0.63	0.26	0.45

^a^significant difference within groups, bold *p*-values indicate significant differences over time

Body weight in high dose reduced significantly from 87.83 (SD 6.09) Kg at W1 to 84.74 (SD 7.00) Kg in W16, whereas in low dose there were no changes (Week 1: 83.12 (SD 9.84) Kg, Week 16: 83.18 (SD 11.68)) Kg. Overall, the changes in weight showed a trend (*p* = 0.06) for significant reduction over 16 weeks, there was also a trend (*p* = 0.06) for Dose*Time interaction. There was reduction in visceral adipose tissue in both the groups (HD W1: 87.08 (39.29) to W16: 79.81 (29.05) vs LD W1: 82.92 (23.72) to W16: 79.16 (21.99)) although not significantly, the overall change showed a trend for reduction over the time of 16 weeks (*p* = 0.06). There were no changes in total subcutaneous adipose tissue in the low dose group. The high dose group showed a reduction in total subcutaneous adipose tissue although this was not significant.

### Changes in glucose and lipid parameters (Table 1)

The fasting glucose levels did not change during the study, however the HbA1c which is a consistent marker of circulating glucose showed a significant reduction in both the doses. The total cholesterol and triglycerides did not change significantly during the study. HDL cholesterol and LDL cholesterol significantly improved over 16 weeks. HDL cholesterol also showed significant improvement within each group (HD W1: 59.39 (12.22) to W16: 73.10 (6.36) vs LD W1: 54.29 (13.71) to W16: 68.21 (8.59).

### Changes in markers associated with inflammation (Table 1)

We measured various markers associated with inflammation such as hsCRP, free fatty acids, Leptin, Adiponectin, sTNFR1, sTNFR2, CD14, TNF-a, IL-6, IL-10 and MCP 1. Amongst these we observed a significant reduction in Leptin indicating that the participants were improving their leptin levels from a range suggestive of obese population to a healthy population [25, 26]. sTNFR1 which is an integral part of signal system in TNF-a mediated inflammation, also showed a significant decrease in 16 weeks.

### Changes in gut microbiota composition (Fig. 1)

**Fig. 1:**
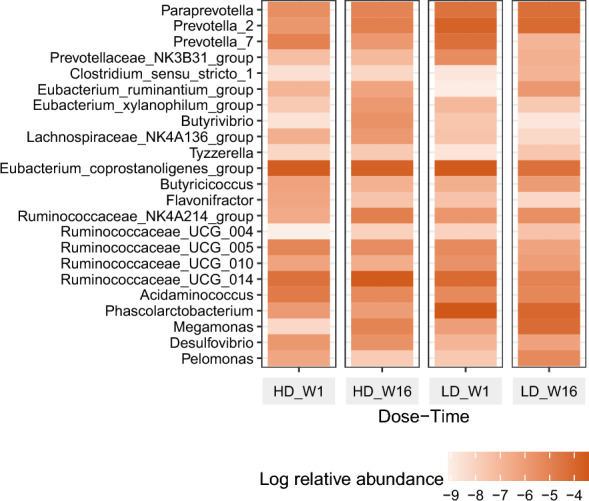
Changes in log relative abundance of microbial species during the study period

After the quality control of the sequences, we obtained average number of 36,984 reads. Removing the OTUs that was below the threshold of prevalence, we obtained 82 genera. Amongst these, we observed various genera to change during the 16 weeks of supplementation. With Dose*Time interaction we noted *Butyricicoccus*, *Eubacterium ruminatium*, *Prevotella* 2 to increase in low dose compared to high dose. Within the low dose *Ruminococcaceae* UCG 004 increased and *Desulfovibrio* decreased from Week 1 to Week 16. Within high dose *Lachnospiraceae* NK4A136 and *Ruminococcaceae* NK4A214 increased significantly. Considering Time response, overall *Phascolarbacterium*, *Desulfovibrio* decreased and *Ruminococcaceae* NK4A214 increased.

### Association between changes gut microbiota and measured variables (Table 2)

**Table 2:** Association between changes in gut microbiota and changes in other measured parameters that varied significantly during the period of intervention

Variable 1	Variable 2	Estimate	Std. Error	t value	p-value
sTNFR1_W1	*Eubacterium xylanophilum group*	− 83.9	18.4	− 4.5	0.0007
HDL-Cholesterol	*Eubacterium coprostanoligenes group*	− 4.8	1.4	− 3.3	0.005
VAT	*Eubacterium xylanophilum group*	− 4.2	1.5	− 2.7	0.017
Body weight	*Clostridium *sensu stricto* 1*	0.79	0.3	2.4	0.029
HbA1c	*Flavonifractor*	0.03	0.01	2.3	0.039
VAT	*Ruminococcaceae UCG 004*	− 2.5	1.1	− 2.189	0.049

We noted that the change in sTNFR1 was negatively associated with changes in *Eubacterium xylanophilum* (*p* < 0.005). The same genera were also associated negatively with changes in visceral adipose tissue (*p* = 0.01). We also observed the increase in *Ruminococcaceae* UCG 004 was associated with decrease in visceral adipose tissue (*p* = 0.04). HDL cholesterol which increased in both the groups was negatively associated with *Eubacterium coprostanoligens*. Additionally, when we tested these associations for metabolites in urine and blood that changed significantly for 16 weeks (Additional file 2 and 3), we noted that change in weight was positively associated to change in colonic metabolites excreted (Additional file 4). The significance was diminished when corrected for multiple p-testing. However, as an exploratory analysis, these values are reported here.

## Discussion

This study reports that supplementation of the polyphenol rich extract Sinetrol^®^ Xpur promotes improvement in HDL, HbA1c, reduction in LDL, and leptin which were time dependent and not dose dependent. The HD (1800 mg/d) consumption tends (*p* = 0.06) to aid weight loss in comparison to the LD (900 mg/d) over a period of 16 weeks. These changes were accompanied with a trend of decrease in visceral adipose tissue. Our study supports the well-established hypothesis of body weight reduction induced by citrus polyphenols as reported in a meta-analysis and dwells deeper into the role of microbiota in these health effects [27]. We observed a time dependent increase in beneficial microbial genera such as *Prevotella* 2, *Ruminococcaceae* NK4A214 and *Eubacterium ruminantium*.

Although previous studies with Sinetrol^®^ Xpur have already indicated a weight loss effect, the dose dependency and the effects on gut microbiota are yet to be explored, thus presented in this study. Contradictory to the previous study with Sinetrol^®^ Xpur we did not observe significant weight loss effects in the LD (900 mg/d). Even though there was no overall weight loss in LD, 3 (out of 10) participants showed a significant reduction of > 1.5 kg (reference set from the meta-analysis [27]), contrarily in the HD group 2 participants showed a weight loss less than 1.5 kg, indicating the potential presence of “responders” and “non-responders” in the study population. Previous studies with flavonoids have also shown the possibility of responders and non-responders depending on their ability to metabolize the compounds. In fact, study by Mollace et al. has shown that increasing the dosage of flavonoid could mitigate the probability of not responding to the flavonoid treatment [28]. Thus, indicating that some non-responders in the LD could benefit from HD which needs to be confirmed with future studies. Another interesting postulation to be regarded while evaluating weight loss studies is that, traditionally weight loss trials also result in decline of lean mass which are metabolically active tissues. Thus, with Sinetrol^®^ Xpur it would be interesting to also understand the body composition while undergoing weight loss. With this understanding we could evaluate if the LD induces fat mass loss without loss in lean mass. Several in vitro and animal studies have explored the mechanism of action of naringenin, hesperidin and its metabolites. Some of these mechanisms are the ability of naringenin, hesperidin and its metabolites to (1) down-regulate oxidative stress (2) inhibit NF-κB activation to suppress inflammatory cytokines and (3) the indirect action of producing colonic metabolites and SCFAs [29–31].

Members of Eubacterium genera are flavonoid degraders which produce butyrate as a by-product. Previous studies with citrus flavonoid supplementation in mice have shown increase in relative abundances of various Eubacterium genera [31, 32]. An important finding from this study is the trend in the reduction of VAT and its relation to the bacterial genera *Eubacterium xylanophilum*. This is consistent with previous report that suggest *Eubacterium xylanophilum* negatively associated with adiposity [33, 34]. Although *Eubacterium xylanophilum* increased only in the HD, we observed other butyrate producing genera such as *Eubacterium ruminatium*, *Prevotella* 2, *Ruminococcaceae* NK4A214 to increase in both the doses. This could indicate that different doses of the supplement could promote different bacterial genera with similar functional capacity of producing butyrate. Butyrate has shown to promote thermogenesis in brown adipose tissue by activating lysine specific demythylase (LSD1) which could be a potential pathway for action of Sinetrol^®^ Xpur.

With Sinetrol^®^ Xpur supplementation, we observed a time dependent increase in resting energy expenditure and carbohydrate expenditure. Interestingly the change in resting energy expenditure of LD was significant. Components of Sinetrol Xpur namely caffeine and flavonoids have been noted for their ability to increase energy expenditure through various mechanism such as inhibition of phosphodiesterase and through stimulation of β2-adrenergic receptors [35]. *Paraprevotella*, a common human gut microbe producing acetic and succinic acid, was associated positively with carbohydrate and resting energy expenditure in our study. Not many reports exist on the relation of *Paraprevotella* and energy expenditure, except the study from Bressa et al., where *Paraprevotella* was noted to be relatively higher in the active women compared to sedentary women [36]. Even though the mechanism of action of *Paraprevotella* on energy expenditure is unknown, we could postulate from previous studies that acetate and succinate could act on upregulation of thermogenesis related genes: peroxisome proliferator-activated receptor-γ (PPARγ) and uncoupling protein 1 (UCP1) [37].

Previous studies have hypothesized that action of flavonoids could be via the phase II and the colonic metabolites in circulation. Predominant of these studies have shown the associations between circulating polyphenolic metabolites and health effects in an acute set up. However, the chronic consumption of polyphenols and excreted metabolites have been controversial [21]. A recent study in mice supplemented with blueberries for 90 days has shown that repeated exposure of polyphenolics could show a nonlinear pattern of urinary excretion of metabolites over time, alongside detection of some metabolites in various tissues [38]. Considering this, an interesting hypothesis we can propose from our study is that with repeated exposure the metabolites are able to accumulate in tissues, thus act in their site of action. Supporting this hypothesis, we observed that colonic urinary metabolites were positively associated with the weight loss. The validity of this hypothesis must be studied in future studies by evaluating the presence of active polyphenol metabolites in the VAT.

Lipid, insulin and adiposity profiles are three closely associated factors determining the metabolic state of an individual. Leptin an important hormone involved in energy regulation was found to be reducing significantly in both the intervention groups. This is consistent with the recent reports that reduction in adipose tissue could also lead to reduction in circulating leptin thus aiding weight loss [39]. Thus, in this study we observe that supplementation of Sinetrol^®^ Xpur irrespective of the dosage lead towards a better metabolic state compared to the baseline. Corresponding to these changes we observe many bacterial genera also moving in same direction within both intervention groups. This could indicate an overall shift in gut microbiota due to Sinetrol^®^ Xpur.

Future studies with measurement of fat composition, fecal SCFAs, metabolites in the tissues and with larger study population could strengthen the hypothesis proposed in this study. A limitation of this study is the absence of control group. However, prior reports on this supplement [19, 40] have shown its efficacy in comparison to placebo. Secondly, even with no significant changes in total energy consumption, there was a significant change in carbohydrate and fat intake over a period of 16 weeks. Thus, even after adjustment for these factors, some residual effects cannot be overruled. Along with the limitations strengths of this study deserve to be mentioned. The inclusion of fat composition data with MRI, metagenomic sequencing of gut microbiota, metabolomic analysis of circulating and excretory metabolites over a period of 16 weeks allowed this study to capture a comprehensive picture of mid-term effect of the polyphenolic extract, Sinetrol^®^ Xpur consumption. Overall, we conclude that Sinetrol^®^ Xpur irrespective of the dosage could benefit the lipid, insulin and visceral adipose metabolism of overweight or obese subjects via modulation of gut microbiota and polyphenol metabolites. Higher dosage of Sinetrol^®^ Xpur could also relatively increase the weight loss compared to the lower dose in a period of 16 weeks.

## Supplementary Information

Additional file1.

## Data Availability

The data presented in this study are available on request from the corresponding author, due to privacy restriction.

## Abbreviations

HD: High dose
LD: Low dose
BIA: Bioelectrical impedance
VAT: Visceral adipose tissue

## References

[1] Pedretti RFE, Hansen D, Ambrosetti M, Back M, Berger T, Ferreira MC, et al. How to optimize the adherence to a guideline-directed medical therapy in the secondary prevention of cardiovascular diseases: a clinical consensus statement from the European Association of Preventive Cardiology. Eur J Prev Cardiol. 2023;30(2):149–66. 10.1093/eurjpc/zwac204. 36098041 10.1093/eurjpc/zwac204

[2] Farhat G, Drummond S, Al-Dujaili EAS. Polyphenols and their role in obesity management: a systematic review of randomized clinical trials. Phytother Res. 2017;31(7):1005–18. 10.1002/ptr.5830. 28493374 10.1002/ptr.5830

[3] Catalkaya G, Venema K, Lucini L, Rocchetti G, Delmas D, Daglia M, et al. Interaction of dietary polyphenols and gut microbiota: microbial metabolism of polyphenols, influence on the gut microbiota, and implications on host health. Food Front. 2020;1(2):109–33. 10.1002/fft2.25.

[4] Chen L, Cao H, Xiao J. 2 - polyphenols: absorption, bioavailability, and metabolomics. In: Galanakis CM, editor. Polyphenols: properties, recovery, and applications. Sawston: Woodhead Publishing; 2018. p. 45–67.

[5] Andres-Lacueva C, Macarulla MT, Rotches-Ribalta M, Boto-Ordóñez M, Urpi-Sarda M, Rodríguez VM, et al. Distribution of resveratrol metabolites in liver, adipose tissue, and skeletal muscle in rats fed different doses of this polyphenol. J Agric Food Chem. 2012;60(19):4833–40. 10.1021/jf3001108. 22533982 10.1021/jf3001108

[6] Janle EM, Lila MA, Grannan M, Wood L, Higgins A, Yousef GG, et al. Pharmacokinetics and tissue distribution of 14C-labeled grape polyphenols in the periphery and the central nervous system following oral administration. J Med Food. 2010;13(4):926–33. 10.1089/jmf.2009.0157. 20673061 10.1089/jmf.2009.0157 PMC3132945

[7] Les F, Carpéné C, Arbonés-Mainar JM, Decaunes P, Valero MS, López V. Pomegranate juice and its main polyphenols exhibit direct effects on amine oxidases from human adipose tissue and inhibit lipid metabolism in adipocytes. J Funct Foods. 2017;33:323–31.

[8] Assini JM, Mulvihill EE, Burke AC, Sutherland BG, Telford DE, Chhoker SS, et al. Naringenin prevents obesity, hepatic steatosis, and glucose intolerance in male mice independent of fibroblast growth factor 21. Endocrinology. 2015;156(6):2087–102. 10.1210/en.2014-2003. 25774553 10.1210/en.2014-2003

[9] Chtourou Y, Fetoui H, Jemai R, ben Slima A, Makni M, Gdoura R. Naringenin reduces cholesterol-induced hepatic inflammation in rats by modulating matrix metalloproteinases-2, 9 via inhibition of nuclear factor κB pathway. Eur J Pharmacol. 2015;746:96–105. 25446569 10.1016/j.ejphar.2014.10.027

[10] Mayneris-Perxachs J, Alcaide-Hidalgo JM, de la Hera E, del Bas JM, Arola L, Caimari A. Supplementation with biscuits enriched with hesperidin and naringenin is associated with an improvement of the Metabolic Syndrome induced by a cafeteria diet in rats. J Funct Foods. 2019;61:103504.

[11] Demonty I, Lin Y, Zebregs YEMP, Vermeer MA, van der Knaap HCM, Jäkel M, et al. The citrus flavonoids hesperidin and naringin do not affect serum cholesterol in moderately hypercholesterolemic men and women. J Nutr. 2010;140(9):1615–20. 10.3945/jn.110.124735. 20660284 10.3945/jn.110.124735

[12] Bolca S, van de Wiele T, Possemiers S. Gut metabotypes govern health effects of dietary polyphenols. Curr Opin Biotechnol. 2013;24(2):220–5. 23040410 10.1016/j.copbio.2012.09.009

[13] Mena P, Ludwig IA, Tomatis VB, Acharjee A, Calani L, Rosi A, et al. Inter-individual variability in the production of flavan-3-ol colonic metabolites: preliminary elucidation of urinary metabotypes. Eur J Nutr. 2019;58(4):1529–43. 10.1007/s00394-018-1683-4. 29616322 10.1007/s00394-018-1683-4

[14] Manach C, Morand C, Gil-Izquierdo A, Bouteloup-Demange C, Rémésy C. Bioavailability in humans of the flavanones hesperidin and narirutin after the ingestion of two doses of orange juice. Eur J Clin Nutr. 2003;57(2):235–42. 12571654 10.1038/sj.ejcn.1601547

[15] Corrêa TAF, Rogero MM, Hassimotto NMA, Lajolo FM. The two-way polyphenols-microbiota interactions and their effects on obesity and related metabolic diseases. Front Nutr. 2019;6:188. 31921881 10.3389/fnut.2019.00188 PMC6933685

[16] Ma G, Chen Y. Polyphenol supplementation benefits human health via gut microbiota: a systematic review via meta-analysis. J Funct Foods. 2020;66:103829.

[17] Cani PD, Neyrinck AM, Fava F, Knauf C, Burcelin RG, Tuohy KM, et al. Selective increases of bifidobacteria in gut microflora improve high-fat-diet-induced diabetes in mice through a mechanism associated with endotoxaemia. Diabetologia. 2007;50(11):2374–83. 10.1007/s00125-007-0791-0. 17823788 10.1007/s00125-007-0791-0

[18] Liao ZL, Zeng BH, Wang W, Li GH, Wu F, Wang L, et al. Impact of the consumption of tea polyphenols on early atherosclerotic lesion formation and intestinal Bifidobacteria in high-fat-fed ApoE−/− mice. Front Nutr. 2016;3:42. 28066771 10.3389/fnut.2016.00042 PMC5175490

[19] Cases J, Romain C, Dallas C, Gerbi A, Rouanet JM. A 12-week randomized double-blind parallel pilot trial of Sinetrol XPur on body weight, abdominal fat, waist circumference, and muscle metabolism in overweight men. Int J Food Sci Nutr. 2015;66(4):471–7. 10.3109/09637486.2015.1042847. 26037199 10.3109/09637486.2015.1042847

[20] Dallas C, Gerbi A, Elbez Y, Caillard P, Zamaria N, Cloarec M. Clinical study to assess the efficacy and safety of a citrus polyphenolic extract of red orange, grapefruit, and orange (Sinetrol-XPur) on weight management and metabolic parameters in healthy overweight individuals. Phytother Res. 2014;28(2):212–8. 10.1002/ptr.4981. 23554029 10.1002/ptr.4981

[21] Muralidharan J, Romain C, Bresciani L, Mena P, Angelino D, Del RD, et al. Nutrikinetics and urinary excretion of phenolic compounds after a 16-week supplementation with a flavanone-rich ingredient. Food Funct. 2023;14(23):10506–19. 37943075 10.1039/d3fo02820h

[22] Brindani N, Mena P, Calani L, Benzie I, Choi SW, Brighenti F, et al. Synthetic and analytical strategies for the quantification of phenyl-γ-valerolactone conjugated metabolites in human urine. Mol Nutr Food Res. 2017;61(9):1700077. 10.1002/mnfr.201700077. 10.1002/mnfr.201700077 28440064

[23] Lluch J, Servant F, Païssé S, Valle C, Valière S, Kuchly C, et al. The characterization of novel tissue microbiota using an optimized 16s metagenomic sequencing pipeline. PLoS One. 2015;10(11):e0142334. 10.1371/journal.pone.0142334. 26544955 10.1371/journal.pone.0142334 PMC4636327

[24] Escudié F, Auer L, Bernard M, Mariadassou M, Cauquil L, Vidal K, et al. FROGS: find, rapidly, OTUs with galaxy solution. Bioinformatics. 2018;34(8):1287–94. 10.1093/bioinformatics/btx791. 29228191 10.1093/bioinformatics/btx791

[25] Gruzdeva O, Borodkina D, Uchasova E, Dyleva Y, Barbarash O. Leptin resistance: underlying mechanisms and diagnosis. Diabetes Metab Syndr Obes. 2019;12:191. 30774404 10.2147/DMSO.S182406 PMC6354688

[26] Owecki M, Nikisch E, Miczke A, Pupek-Musialik D, Sowiski J. Leptin, soluble leptin receptors, free leptin index, and their relationship with insulin resistance and BMI: high normal BMI is the threshold for serum leptin increase in humans. Horm Metab Res. 2010;42(8):585–9. 10.1055/s-0030-1253422. 20455195 10.1055/s-0030-1253422

[27] Wang X, Li D, Liu F, Cui Y, Li X. Dietary citrus and/or its extracts intake contributed to weight control: evidence from a systematic review and meta-analysis of 13 randomized clinical trials. Phytother Res. 2020;34(8):2006–22. 10.1002/ptr.6673. 32182635 10.1002/ptr.6673

[28] Mollace V, Sacco I, Janda E, Malara C, Ventrice D, Colica C, et al. Hypolipemic and hypoglycaemic activity of bergamot polyphenols: from animal models to human studies. Fitoterapia. 2011;82(3):309–16. 21056640 10.1016/j.fitote.2010.10.014

[29] Ren B, Qin W, Wu F, Wang S, Pan C, Wang L, et al. Apigenin and naringenin regulate glucose and lipid metabolism, and ameliorate vascular dysfunction in type 2 diabetic rats. Eur J Pharmacol. 2016;773:13–23. 26801071 10.1016/j.ejphar.2016.01.002

[30] Jung UJ, Lee MK, Park YB, Kang MA, Choi MS. Effect of citrus flavonoids on lipid metabolism and glucose-regulating enzyme mRNA levels in type-2 diabetic mice. Int J Biochem Cell Biol. 2006;38(7):1134–45. 16427799 10.1016/j.biocel.2005.12.002

[31] Wang F, Zhao C, Yang M, Zhang L, Wei R, Meng K, et al. Four citrus flavanones exert atherosclerosis alleviation effects in ApoE–/– mice via different metabolic and signaling pathways. J Agric Food Chem. 2021;69(17):5226–37. 10.1021/acs.jafc.1c01463. 33890787 10.1021/acs.jafc.1c01463

[32] Sost MM, Ahles S, Verhoeven J, Verbruggen S, Stevens Y, Venema K. A citrus fruit extract high in polyphenols beneficially modulates the gut microbiota of healthy human volunteers in a validated in vitro model of the colon. Nutrients. 2021;13(11):3915. 34836169 10.3390/nu13113915 PMC8619629

[33] Lozano CP, Wilkens LR, Shvetsov YB, Maskarinec G, Park SY, Shepherd JA, et al. Associations of the dietary inflammatory index with total adiposity and ectopic fat through the gut microbiota, LPS, and C-reactive protein in the multiethnic cohort-adiposity phenotype study. Am J Clin Nutr. 2022;115(5):1344–56. 10.1093/ajcn/nqab398. 34871345 10.1093/ajcn/nqab398 PMC9071464

[34] Wei J, Zhao Y, Zhou C, Zhao Q, Zhong H, Zhu X, et al. Dietary polysaccharide from Enteromorpha clathrata attenuates obesity and increases the intestinal abundance of butyrate-producing bacterium, *Eubacterium xylanophilum*, in mice fed a high-fat diet. Polymers (Basel). 2021;13(19):3286. 34641102 10.3390/polym13193286 PMC8512240

[35] Vaughan RA, Conn CA, Mermier CM. Effects of commercially available dietary supplements on resting energy expenditure: a brief report. ISRN Nutr. 2014;2014:650264. 10.1155/2014/650264. 24967272 10.1155/2014/650264 PMC4045300

[36] Bressa C, Bailén-Andrino M, Pérez-Santiago J, González-Soltero R, Pérez M, Montalvo-Lominchar MG, et al. Differences in gut microbiota profile between women with active lifestyle and sedentary women. PLoS One. 2017;12(2):e0171352. 10.1371/journal.pone.0171352. 28187199 10.1371/journal.pone.0171352 PMC5302835

[37] Canfora EE, Van Der Beek CM, Jocken JWE, Goossens GH, Holst JJ, Olde Damink SWM, et al. Colonic infusions of short-chain fatty acid mixtures promote energy metabolism in overweight/obese men: a randomized crossover trial. Sci Rep. 2017;7(1):1–12. 28539646 10.1038/s41598-017-02546-x PMC5443817

[38] Cladis DP, Simpson AMR, Cooper KJ, Nakatsu CH, Ferruzzi MG, Weaver CM. Blueberry polyphenols alter gut microbiota & phenolic metabolism in rats. Food Funct. 2021;12(6):2442–56. 33629093 10.1039/d0fo03457f PMC8011555

[39] Hankir MK, Seyfried F. Partial leptin reduction: an emerging weight loss paradigm. Trends Endocrinol Metab. 2020;31(6):395–7. 32396841 10.1016/j.tem.2020.03.001

[40] Park SJ, Sharma A, Bae MH, Sung HC, Kim NK, Sung E, et al. Efficacy and safety of Sinetrol-XPur on weight and body fat reduction in overweight or obese adults: a 12-week, randomized, double-blind, parallel, placebo-controlled trial. J Med Food. 2020;23(3):335–42. 10.1089/jmf.2019.4649. 32130058 10.1089/jmf.2019.4649

